# Assessment of Small Bowel Motility and SMA Blood Flow Studied with Transabdominal Ultrasound

**DOI:** 10.1055/a-1925-1893

**Published:** 2022-09-23

**Authors:** Kim Nylund, Andreas Jessen Gjengstø, Hilde Løland von Volkmann, Odd Helge Gilja

**Affiliations:** 1 Department of Medicine, Haukeland University Hospital, Bergen, Norway; 2 Department of Clinical Medicine, University of Bergen, Bergen, Norway; 3 National Center of Ultrasound in Gastroenterology, Haukeland University Hospital, Bergen, Norway

**Keywords:** small bowel, blood flow, motility, THEMES, gastrointestinal ultrasound, healthy volunteers

## Abstract

**Purpose** Gastrointestinal ultrasound (GIUS) is a noninvasive imaging technique that may be used to study physiological changes in the small bowel. The aim of the study was to investigate the feasibility of measuring blood flow (BF) in the superior mesenteric artery (SMA) and regional motility in the small bowel with GIUS before and after a test meal and to compare ultrasound parameters to demographic factors such as age, sex, height, weight, and smoking habits.

**Materials and Methods** 122 healthy volunteers aged 20 to 80 were examined after an overnight fast. Small bowel motility was registered in the upper left and lower right quadrants (ULQ and LRQ) with TUS and BF in the SMA with pulsed wave Doppler. The first 23 volunteers also received a 300 Kcal test meal and were re-examined 30 min postprandial.

**Results** The feasibility of measuring BF was 97% in fasting patients while motility could be detected in 52% and 62% in the ULQ and LRQ, respectively. Females had a lower resistive index (RI) and a higher mean velocity than males, while the overall BF correlated with height. The RI had a negative correlation with age. Healthy volunteers with motility in the ileum were on average younger than those without motility. After the test meal, motility could be detected in the ULQ and LRQ in 95% and 90%, respectively, and the mean number of contractions in the ULQ increased significantly. As expected, there was a clear increase in all BF-parameters postprandially.

**Conclusion** Regional motility in the small bowel was easier to detect after a test meal. There were some associations between demographic parameters and ultrasound parameters but overall the effects were relatively small.

## Introduction

Pathological changes due to disease are often related to structural changes but may also cause changes in physiology. In small bowel disease, motility changes occur in celiac disease and bowel obstruction, and changes in blood flow are features of inflammatory bowel disease and abdominal angina 1 2 3 . Small bowel physiology is difficult to study, however, as the small bowel is long and difficult to reach with instruments. Also, introducing an instrument into the lumen of the small bowel may change the very phenomenon you want to study.

Regional effects of peristalsis can be studied using manometry 4 , while transition times can be investigated using breath tests and scintigraphy 5 . Wireless motility capsules provide data both on local pressure and transit times, but the link between pressure and actual peristaltic movement is not established as many factors other than peristalsis can register as pressure. Manometry is invasive, difficult to interpret, and not readily available. Breath tests for measuring transit time are problematic as the test in itself affects transit and small bacterial overgrowth complicates interpretation 6 . Scintigraphy methods are poorly standardized with a wide range of normal values, and gastric and colonic transition will affect the results in the small bowel. Wireless motility capsules depend on pH landmarks which are not present in 5–10% of patients 5 . It would thus seem like none of the current methods are ideal 5 6 .

Cross-sectional imaging offers another window to the small bowel where it is theoretically possible to study the normal behavior of the organ such as motility and blood flow. In the last 10–15 years dynamic magnetic resonance imaging (MRI) or cine-MRI has been used to study both regional and global motility in the small bowel and dysmotility in different disease states 7 . MRI is, however, an expensive tool with limited availability.

In comparison, gastrointestinal ultrasound (GIUS) is considerably cheaper and far more readily available. GIUS can more than rival MRI with regards to temporal and spatial resolution as a standard 5 MHz abdominal probe has an axial and lateral resolution <0.5 mm and a frame rate >30 Hz in B-mode 8 . GIUS is limited to the study of regional motility, however, as only a section of the bowel can be studied over time. Another useful aspect of ultrasound is that it can be used to investigate bowel blood flow using Doppler during the same session as motility 9 . Methodologically, we plan to expand on our current knowledge and experience with upper GI motility using ultrasonography 10 11 12 .

Currently, the literature on bowel motility using ultrasound is limited. Early studies indicate a use for investigating motility in patients with celiac disease 2 13 14 15 . In these studies, they only registered the presence or absence of peristalsis in fasting patients 2 16 . In a study by von Volkmann et al. on patients with a defect in the *GUCY2C* gene causing familial *GUCY2C* -diarroea syndrome (FGDS), regional motility in the jejunum and terminal ileum was investigated using transabdominal ultrasound. They registered whether contractions in the small bowel were occlusive or non-occlusive as well as the presence of “back and forth” movement 12 . In another study using a meal challenge, they again compared the FGDS group with healthy volunteers and found that the patients had significantly more non-occlusive contractions in the ileum after the meal challenge 11 . Non-occlusive contractions are not a finding specific for FGDS patients and have been reported in prestenotic bowel segments as well 17 .

The blood flow to the small intestine is mostly through the superior mesenteric artery. As early as 1998, Moneta et al. studied meal effects in healthy volunteers and found a clear increase in all flow parameters after 30 minutes 18 . Similar studies with slightly different setups such as changes in meal composition or parameters being investigated have been performed 19 20 . Results suggest that a fatty meal causes the largest flow increase 18 21 , but it may be delayed due to differences in gastric emptying. The delay can be avoided by giving the meal directly into the duodenum 22 . Even the type of fatty acids used in the fatty meal can have different effects on the flow. Long chain fatty acids (12–18 carbons) cause the largest increase 23 . On the other hand, parenteral nutrition lowers the flow in the SMA 24 .

Frequently, small bowel motility and blood flow parameters are included in studies of pathology without there being clear data on what the normal range of these data are. Typically, a matched control group is used 1 13 14 15 19 25 . This may be useful when trying to compare a specific population subgroup with its counterpart but cannot be extrapolated to larger cohorts. A study including a large healthy population with a wide range in age, balanced for gender and no BMI restrictions should give results that can be generalized to larger populations.

The aim of the study was to measure blood flow in the superior mesenteric artery and regional motility in the small bowel with transabdominal ultrasound. A further aim was to investigate how ultrasound parameters related to physiological measurements such as motility and how arterial blood flow was affected by fasting state, age, sex, weight, height, and smoking status.

## Materials and methods

### Study subjects

122 healthy volunteers (61 men, 61 women) aged 20–80 were recruited. The participants were divided into six subgroups according to age with 10 from each gender in each subgroup: 20–29, 30–39, 40–49, 50–59, 60–69, and 70–79 years. The subjects were recruited from the hospital staff or among retirees. The exclusion criteria were known GI disease, previous surgery of the GI tract except appendectomy, symptoms that could be related to the gastrointestinal tract within the last two weeks, known heart failure, medication with a known or suspected influence on the GI tract, and pregnancy. The study was approved by the Regional Ethics Committee of Western Norway and informed consent was obtained from all participants before entering the study. Background information including age, sex, weight, height, and smoking status was recorded.

The first 23 volunteers recruited were examined with GIUS both before and after a test meal and the same parameters were registered in the first and second part of the examination. A 200 ml test meal containing 300 Kcal (Nutricia Norge AS, Oslo, Norway) was ingested over a 5-minute period. The meal contained a mixture of 24.6 g of carbohydrates, 13.4 g of fat and 20 g of protein. The volunteer rested in a supine position for 30 minutes after the meal before the second part of the examination.

### Gastrointestinal ultrasound examination

The examination was performed after overnight fasting (> 8 hours). The healthy subjects were in a supine position and were asked to rest for 5 minutes on a bench before starting the examination. We used an ultrasound scanner (GE Logiq 9, GE Healthcare, Milwaukee, USA) equipped with two ultrasound transducers: a curvilinear 1.5–4.5 MHz transducer (4 C) and a linear 6–8 MHz transducer (9 L). The 4 C was set to center frequencies 4 MHz and the 9 L to 8 MHz.

**Fig 1:**
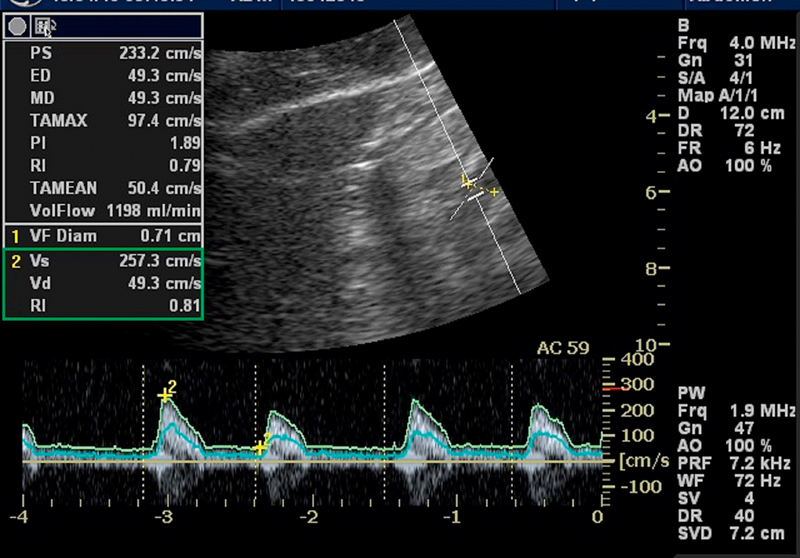
An example of flow measurement in the superior mesenteric artery using spectral Doppler.

The flow in the superior mesenteric artery (SMA) was measured using pulse wave Doppler with the 4 C transducer with a center frequency of 4 MHz and a Doppler frequency of 1.9 MHz. The measurement was performed during breath-hold under normal inspiration. The SMA was identified in a midline section and the sample volume was placed at least 1 cm from where the artery branches from the aorta to avoid turbulence. The sample volume was adjusted to fit the diameter of the superior mesenteric artery. The automated tracing over an average of 3 heartbeats was used for quantifying the time-averaged mean needed to calculate flow while the actual diameter was measured in B-mode to improve the accuracy of cursor placement. The maximum and minimum velocities used for calculating the resistive index and pulsatile index were measured manually in the spectral curve to increase accuracy ( Fig. 1 ).

**Fig 2:**
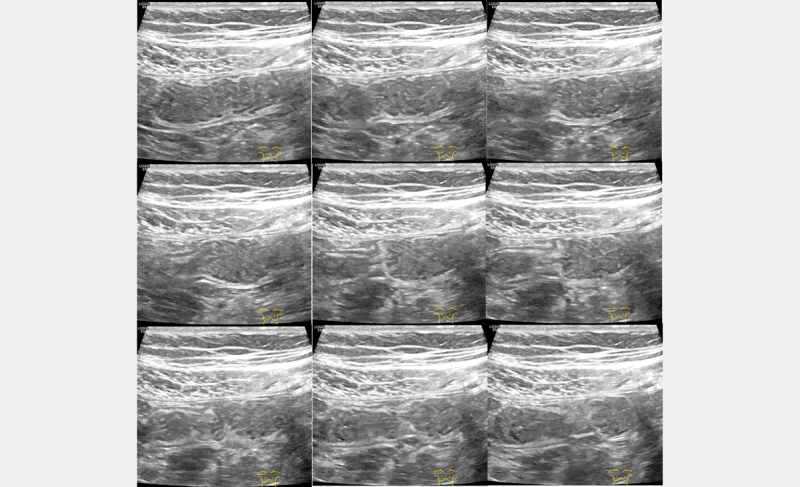
A contraction propagating through a longitudinal section of a jejunal loop is shown. Nine sequential images starting from the upper left corner and going from left to right demonstrate how a contraction starts from the left and propagates to the right. In the study this was registered as a single contraction.

Small bowel motility was registered in two regions in the abdomen with the 9 L transducer, the upper left quadrant and the lower right quadrant. A small bowel segment was identified in each region and observed over a period of 1 min. A segment with visible peristalsis was chosen over a segment without motility, and if there were several segments with visible motility, the segment that seemed most active at the start of the registration was selected for acquisition. The segment was observed with the ultrasound imaging plane through the longitudinal axis of the bowel. Although some time was spent searching for the bowel segment to observe, this period never lasted more than 2 minutes per segment. The bowel segment in the upper left quadrant was defined as the jejunum and the bowel segment in the lower right segment as the ileum. A peristaltic contraction was defined as any active propagating narrowing of the lumen ( Fig. 2 ). The number of observed peristaltic contractions was assessed in the observation period.

### Statistics

Comparison between groups of healthy volunteers was performed using the Fischer exact test, Student’s T-test, or Mann-Whitney U test for paired samples, and comparisons between healthy volunteers before and after the test meal were performed using the Student’s T-test for paired samples or the Wilcoxon signed-rank test for continuous variables. Correlations were done using Pearson’s correlation coefficient for continuous variables. All statistical analyses were performed using SPSS software version 26 (IBM Inc., Armonk, NY, USA).

## Results

There were no exclusions due to an inability of the volunteer to ingest the test meal. In three patients, one of whom was fasting and two of whom received a test meal, motility was not registered due to a failure to save the cine loops correctly. In 4 subjects flow could not be measured due to poor visualization of the SMA. In some cases not all the parameters were registered due to body habitus or technical errors. There were no differences between the meal group and non-meal group except for age ( Table 1 ).

**Table:** **Table 1** Comparison between meal group and non-meal group in a study using gastrointestinal ultrasound. The mean*is displayed with the standard deviation and the median ** with the range in parentheses. For sex the number of males and females is shown.

Variable	Meal group N=23	Non-meal group N=99
Age (years)*	40.6 (14.3)	50.2 (17.2)
Weight (kg)*	73.0 (11.5)	74.8 (13.7)
Height (M)*	1.75 (0.08)	1.73 (0.08)
Sex (F/M)	8/15	53/46
Smoking	3/23	12/99
Package years **	6 (9.5)	4.5 (56.3)

### General motility

The motility was registered in 121 healthy volunteers in the fasting state. 46/121 (38%) had no peristaltic movements in the fasting state in the upper left quadrant, while the corresponding number was 50/121 (41%) in the lower right quadrant. The overall median number of contractions was 4/min in both the jejunum and the ileum and the interquartile range (IQR) was 8/min. If healthy volunteers without any peristalsis were excluded from the analysis, the median number of contractions was 7/min (IQR=6) in the jejunum and 6/min (IQR=6) in the ileum.

### Age, sex, and motility

Healthy volunteers with no fasting motility in the ileum were on average older than those with motility (53.3 (17.7) years vs. 45.0 (15.9) years, p=0.008). This was not significant in the jejunum, and there were no significant correlations in the number of peristaltic contractions with regard to age, sex, weight, or height in the jejunum or the ileum for the subgroup of volunteers with detectable motility during fasting.

### Postprandial motility

**Fig 3:**
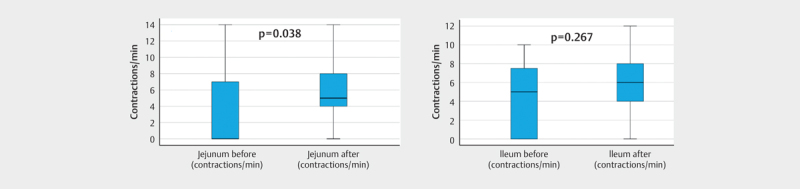
Boxplots of the number of contractions per min in the jejunum and ileum assessed by ultrasound before and after the test meal. Using a paired Wilcoxon signed-rank test, there was a significant difference in the number of contractions in the jejunum, but not in the ileum (p-values shown in image). The central black line is the median, the boxes are the upper and lower quartiles, and the whiskers are the 10th and 90th percentiles.

Motility was present during fasting in 11/21 (52%) in the upper left and 13/21(62%) in the lower right quadrant. After the meal, the corresponding rate of detection was 20/21 (95%) and 19/21 (90%). In this group the median number of contractions in the upper left quadrant was 0 (IQR=7) before the meal and 5 (IQR=4) after the meal. In the lower right quadrant, the median was 5 (IQR=9) before the meal and 6 (IQR=4) after the meal ( Fig. 3 ).

### Blood flow overall

In 118 of 122 healthy volunteers, the Doppler flow in the SMA was measured. None were excluded due to inability of the operator to find an acceptable Doppler angle (<60 degrees). Accordingly, the feasibility was 97%. The blood flow measurements for the whole group are shown in Table 2 .

**Table:** **Table 2** Flow parameters in the superior mesenteric artery in fasting volunteers assessed by Doppler ultrasound

Parameter	N	Lowest	Highest	Mean	Std. deviation
Max velocity (cm/s)	118	74.5	292.3	158	45.1
Mean velocity (cm/s)	118	16.0	52.3	29.8	8.4
Mean flow (ml/min)	118	166.3	1188.3	554.7	228.9
Resistive Index (RI)	118	0.67	0.85	0.78	0.04
Diameter (mm)	118	4.1	9.0	6.3	1.0

### Age, sex, and blood flow

There was a negative correlation between age and resistive index (r= –0.2, p=0.03). It did not seem to affect the other flow parameters. The height correlated both with the diameter of the SMA (r=0.52, p<0.001) and blood flow (r=0.34, p<0.001), but also with the resistive index (r=0.2, p=0.033). Females had on average a higher mean velocity in the SMA (31.6 ± 9.2 cm/s vs 28.21 ± 7.3 cm/s, p= 0.023) and lower RI than men (RI=0.77 0.4 vs RI 0.78 0.4, p=0.041).

### Weight, height, BMI, smoking, and blood flow

There was a correlation both between mean blood flow (r=0.30, p=0.001) and resistive index (r=0.48, p=0.20) for patient height, but not for mean velocity (r=–0.12, p=0.181). There were only 15 current smokers in the cohort and there were no differences in any of the flow parameters between smokers and non-smokers.

### Meal and blood flow

**Fig 4:**
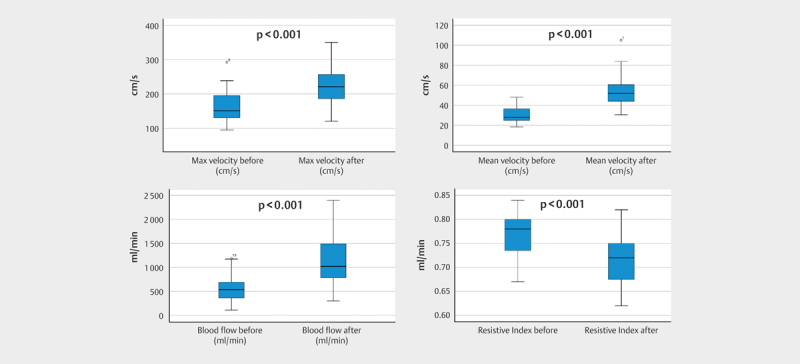
Boxplots of maximum velocity, mean velocity blood flow, and resistive index in 23 healthy volunteers measured by Doppler ultrasound before and after test meal. Using a paired Student's T-test, the measurements are all significantly different with p<0.001. The central black line is the median, the boxes are the upper and lower quartiles, and the whiskers are the 10th and 90th percentiles.

In all 23 volunteers receiving the test meal, the flow in the SMA could be measured. The results are summarized in Table 3 . There were significant changes in the flow parameters. On average, the maximum velocity increased from 161.5 cm/s to 229.7 cm/s, the mean blood flow from 562.2 cm/s to 1132.3 cm/s and the RI decreased from 0.77 to 0.72. Comparison of the different parameters in these 23 volunteers is shown as boxplots in Fig 4 .

**Table:** **Table 3** Flow parameters evaluated by Doppler ultrasound of the superior mesenteric artery in 23 healthy volunteers after the test meal

Parameter	N	Minimum	Maximum	Mean	Std. deviation
Max velocity (cm/s)	23	120.0	350.4	229.7	65.9
Mean velocity (cm/s)	23	30.6	105.0	54.6	17.2
Mean flow (ml/min)	23	302.1	2401.3	1132.3	511.2
Resistive Index (RI)	23	0.62	0.82	0.72	0.05
Diameter (mm)	23	6.5	4.5	9.0	1.1

## Discussion

This study shows the importance of a test meal when studying small bowel motility using ultrasound. As expected, the detection of regional motility increased markedly after a meal challenge. Moreover, we also found that healthy volunteers with no detected fasting motility in the ileum were on average older than those with detected motility. There was excellent feasibility for measuring flow in the SMA both fasting and postprandially. There was also a clear increase in SMA blood flow after the meal, while there was only an increase in the number of peristaltic contractions in the jejunum after the meal. The study indicated some relationships between different demographic factors, flow in the SMA, and measurements of motility. There was increased flow in the SMA in relation to height and an inverse relation between age and RI. Furthermore, the mean velocity was slightly higher and the RI was slightly lower in the SMA in women.

Our findings with regard to fasting blood flow in the SMA correspond to the existing literature except for the resistive index which on average we found to be slightly lower. While we found a mean of 0.78, Dietrich et al. in 2007 in a literature review showed that the mean varied between 0.89 and 0.81 1 . However, none of these studies had more than 50 participants and most were examined as controls in case-control studies. 40 patients over the age of 60 years were included in our study which is uncommon in standard control groups. As there was an inverse relationship between age and resistive index, this may explain some of the differences in RI between our study and previous studies. Another explanation can be that the sample volume was fitted to the diameter of the vessel. The size of the sample volume is rarely reported 18 19 20 21 , but if a sample volume is smaller than the vessel diameter, it will not include the full velocity spectrum. Symersky et al. set the sample area in similar fashion and found a fasting RI of 0.80 (±0.03), which is comparable to our results 22 . For the other parameters such as peak systolic velocity (81–147 cm/s), mean velocity (16–40 cm/s), and blood flow (305–639 ml/min), our values were comparable to previous published literature 1 .

There is considerable variability in the flow measurements in the SMA. Especially the blood flow varies greatly with a range of 166.3 to 1188.3 ml/min. This is also suggested in previous studies 1 25 . This is partly due to differences in patient height which clearly affects the diameter of the SMA and hence the blood flow. Some of the variability can be explained by differences in patient size, but not all. There may, for instance, be individual differences in how much of the bowel is supplied by the SMA.

The higher mean velocity in the SMA in women has been previously reported by Szinnai et al. in 2001 although they found a larger difference than in our study (47 vs. 39 cm/s) 26 . While we found a lower RI in women, they measured and found a lower PI. There were no significant differences in the peak velocity. Overall, the results suggest that women on average have a slightly lower resistance in their splanchnic circulation when fasting.

The comparison of postprandial blood flow is more difficult to compare with existing literature since the meal used, administration method, and time of investigation will often vary between studies 21 22 26 . In these studies, the investigators found a clear drop of 0.05–0.1 in RI 30 minutes after the meal and an average increase in blood flow from 50% to 150%. This is in line with the results from this study.

During fasting the migrating motor complex is responsible for the motility in the small bowel. It consists of 3 phases where the longest is phase I in which there is no motility. Typically, this phase can last for two hours interrupted by about 15 min of irregular contractions during phase II and regular phasic contractions for 5 min in phase III 27 28 . During phase III the peristaltic movements propagate through the whole length of the small bowel 29 . During fasting the small bowel is mostly in phase I and thus no motility can be detected. If only phases II and III give rise to contractions, we should only have detected motility in about 20% of our healthy volunteers during an observation time that was less than 10 min in total. This could be caused by the delay in propagation of peristalsis from the upper GI tract. Since the speed of propagation slows down in the distal direction, this causes the length of phases II and III to increase in the distal part of the small intestine 29 .

The frequency of contractions in the jejunum was higher postprandially while this was not the case for the ileum. This is probably because the 2 ^nd^ investigation was done after only 30 min which would prevent the chyme from reaching the ileum. In this study, healthy volunteers that had no motility were older on average. The number of contractions did not seem to correlate with age. This may suggest that older individuals spend a longer time in phase I. This contradicts previous findings with manometry in the upper part of the small bowel where no indication of a senescence of phase 1 of MMC was found 30 31 . Our findings are only suggestive, and we clearly need a more precise method and longer observation time than we have employed in this study to obtain more robust results regarding the MMC patterns. However, to the advantage of GIUS, it can be used to study motility at several different locations not available for manometry.

Our study had some limitations. The meal group was on average younger than the fasting group due to differences in recruitment. This may have affected the results to some degree as we did find some effects of age on parameters such as RI and presence of fasting motility. Furthermore, the meal group was too small to fully ascertain the relationship between ultrasound measurements and demographic factors. Although the differences we found in the fasting group related to age were small, it does not necessarily mean that this will also be the case after a test meal.

We did not investigate the variability in assessing motility and it can be argued that the method for selecting a bowel segment for studying motility was biased and inaccurate as the bowel segment was chosen purely based on visual inspection. However, the purpose of the study was to investigate *regional* motility and not to investigate if it is representative for the entire small bowel.

## Conclusion

We successfully measured blood flow in the SMA with pulse wave Doppler before and after a test meal in almost all subjects, and we found distinct changes in response to a meal. There is a considerable overlap between the parameters before and after the meal suggesting that there is variability caused by other factors not investigated in this study. The study of motility cannot be done without taking fasting state into account. While disease states probably can cause pathological changes both in the migrating motor complex and in the meal-induced motility, the former may be difficult to investigate with GIUS since frequently no motility is detected during fasting. A test meal increases intestinal motility as observed by gastrointestinal ultrasonography. Further development of ultrasound methods using a test meal in combination with luminal contrast to improve characterization of regional motility may improve this technique.

## References

[1] Dietrich C F Jedrzejczyk M Ignee A Sonographic assessment of splanchnic arteries and the bowel wall Eur J Radiol 2007 64 202 212 17923366 10.1016/j.ejrad.2007.06.034

[2] Fraquelli M Colli A Colucci A Accuracy of ultrasonography in predicting celiac disease Arch Intern Med 2004 164 169 174 14744840 10.1001/archinte.164.2.169

[3] Hollerweger A Maconi G Ripolles T Gastrointestinal Ultrasound (GIUS) in Intestinal Emergencies - An EFSUMB Position Paper Ultraschall Med 2020 41 646 657 10.1055/a-1147-1295 32311749

[4] Stanghellini V Cogliandro R Cogliandro L Clinical use of manometry for the diagnosis of intestinal motor abnormalities Dig Liver Dis 2000 32 532 541 11057929 10.1016/s1590-8658(00)80011-0

[5] Rao S S Camilleri M Hasler W L Evaluation of gastrointestinal transit in clinical practice: position paper of the American and European Neurogastroenterology and Motility Societies Neurogastroenterol Motil 2011 23 8 23 21138500 10.1111/j.1365-2982.2010.01612.x

[6] Lee A Baker J Hasler WL GI Motility Testing: Stomach, Small Bowel, and Colon J Clin Gastroenterol 2019 53 159 169 30702487 10.1097/MCG.0000000000001138

[7] de Jonge C S Smout A Nederveen A J Evaluation of gastrointestinal motility with MRI: Advances, challenges and opportunities Neurogastroenterol Motil 2018 30 10.1111/nmo.13257 29265641

[8] Chou YHFB Shen C Basics of ultrasonography. In: Nürnberg D CM, Gilja OH, Sporea I, Sirli R ed, WFUMB Course Book. 1 ed World Federation for Ultrasound in Medicine and Biology 2021 1 14

[9] Nylund K Maconi G Hollerweger A EFSUMB Recommendations and Guidelines for Gastrointestinal Ultrasound Ultraschall Med 2017 38 e1 e15 27604052 10.1055/s-0042-115853

[10] Gilja O H Hausken T Wilhelmsen I Impaired accommodation of proximal stomach to a meal in functional dyspepsia Dig Dis Sci 1996 41 689 696 8674389 10.1007/BF02213124

[11] von Volkmann H L Bronstad I Gilja O H Prolonged intestinal transit and diarrhea in patients with an activating GUCY2C mutation PLoS One 2017 12 e0185496 28957388 10.1371/journal.pone.0185496 PMC5619782

[12] von Volkmann H L Nylund K Tronstad R R An activating gucy2c mutation causes impaired contractility and fluid stagnation in the small bowel Scand J Gastroenterol 2016 51 1308 1315 27338166 10.1080/00365521.2016.1200139

[13] Dietrich C F Brunner V Seifert H [Intestinal B-mode sonography in patients with endemic sprue. Intestinal sonography in endemic sprue] Ultraschall Med 1999 20 242 247 10670069 10.1055/s-1999-8921

[14] Micetic-Turk D Umek-Bradac S Dolinsek J Ultrasonographic assessment of celiac disease in children: comparison with antiendomysium antibodies and histology Wien Klin Wochenschr 2001 113Suppl 327 31 15503617

[15] Rettenbacher T Hollerweger A Macheiner P Adult celiac disease: US signs Radiology 1999 211 389 394 10228518 10.1148/radiology.211.2.r99ma39389

[16] Fraquelli M Sciola V Villa C The role of ultrasonography in patients with celiac disease World J Gastroenterol 2006 12 1001 1004 16534837 10.3748/wjg.v12.i7.1001 PMC4087888

[17] Hollerweger A Wustner M Dirks K Bowel Obstruction: Sonographic Evaluation Ultraschall Med 2015 36 216 235quiz 236-218 25905814 10.1055/s-0034-1399292

[18] Moneta G L Taylor D C Helton W S Duplex ultrasound measurement of postprandial intestinal blood flow: effect of meal composition Gastroenterology 1988 95 1294 1301 3049214 10.1016/0016-5085(88)90364-2

[19] Giovagnorio F Diacinti D Vernia P Doppler sonography of the superior mesenteric artery in Crohn's disease AJR Am J Roentgenol 1998 170 123 126 9423614 10.2214/ajr.170.1.9423614

[20] Ludwig D Wiener S Bruning A Mesenteric blood flow is related to disease activity and risk of relapse in Crohn's disease: a prospective follow-up study Am J Gastroenterol 1999 94 2942 2950 10520849 10.1111/j.1572-0241.1999.01442.x

[21] Sidery M B Macdonald I A Blackshaw PE Superior mesenteric artery blood flow and gastric emptying in humans and the differential effects of high fat and high carbohydrate meals Gut 1994 35 186 190 8307468 10.1136/gut.35.2.186 PMC1374492

[22] Symersky T Huisman E J Wasser M N Effect of fat digestion on superior mesenteric artery blood flow in humans Clin Physiol Funct Imaging 2007 27 7 11 17204031 10.1111/j.1475-097X.2007.00711.x

[23] Vu M K Berkhoudt J Van Oostayen J A Effect of triglycerides with different fatty acid chain length on superior mesenteric artery blood flow Acta Physiol Scand 2001 171 37 41 11350261 10.1046/j.1365-201X.2001.00778.x

[24] Gatt M MacFie J Anderson A D Changes in superior mesenteric artery blood flow after oral, enteral, and parenteral feeding in humans Crit Care Med 2009 37 171 176 19050615 10.1097/CCM.0b013e318192fb44

[25] Sjekavica I Barbaric-Babic V Krznaric Z Assessment of Crohn's disease activity by doppler ultrasound of superior mesenteric artery and mural arteries in thickened bowel wall: cross-sectional study Croat Med J 2007 48 822 830 18074417 10.3325/cmj.2007.6.822 PMC2213815

[26] Szinnai C Mottet C Gutzwiller J P Role of gender upon basal and postprandial systemic and splanchnic haemodynamics in humans Scand J Gastroenterol 2001 36 540 544 11346210 10.1080/003655201750153458

[27] Husebye E The patterns of small bowel motility: physiology and implications in organic disease and functional disorders Neurogastroenterol Motil 1999 11 141 161 10354340 10.1046/j.1365-2982.1999.00147.x

[28] Husebye E Skar V Aalen O O Digital ambulatory manometry of the small intestine in healthy adults. Estimates of variation within and between individuals and statistical management of incomplete MMC periods Dig Dis Sci 1990 35 1057 1065 2390920 10.1007/BF01537575

[29] Deloose E Janssen P Depoortere I The migrating motor complex: control mechanisms and its role in health and disease Nat Rev Gastroenterol Hepatol 2012 9 271 285 22450306 10.1038/nrgastro.2012.57

[30] Anuras S Sutherland J Small intestinal manometry in healthy elderly subjects J Am Geriatr Soc 1984 32 581 583 6747169 10.1111/j.1532-5415.1984.tb06136.x

[31] Husebye E Engedal K The patterns of motility are maintained in the human small intestine throughout the process of aging Scand J Gastroenterol 1992 27 397 404 1529275 10.3109/00365529209000095

